# Incidence and risk factors of preoperative deep vein thrombosis in patients with intertrochanteric fractures: a retrospective study

**DOI:** 10.1186/s13018-022-03268-1

**Published:** 2022-08-03

**Authors:** Kai Song, Bowen Zhu, Yao Yao, Qing Jiang, Jin Xiong, Hongfei Shi

**Affiliations:** 1 State Key Laboratory of Pharmaceutical Biotechnology, Division of Sports Medicine and Adult Reconstructive Surgery, Department of Orthopedic Surgery, Nanjing Drum Tower Hospital, The Affiliated Hospital of Nanjing University Medical School, 321 Zhongshan Road, Nanjing, 210008 Jiangsu People’s Republic of China; 2 Laboratory for Bone and Joint Disease, Model Animal Research Center (MARC), Nanjing University, Nanjing, 210093 Jiangsu People’s Republic of China; 3 Department of Orthopedic Surgery, Nanjing Drum Tower Hospital, The Affiliated Hospital of Nanjing University Medical School, 321 Zhongshan Road, Nanjing, 210008 Jiangsu People’s Republic of China

**Keywords:** Intertrochanteric fracture, Deep vein thrombosis, Epidemiology, Risk factors

## Abstract

**Background:**

The risk of perioperative complications remains high in patients with intertrochanteric fractures. Immobilization after injury may predispose these patients to deep vein thrombosis (DVT) while waiting for surgery. The aims of this study were to determine the incidence of preoperative DVT in patients with intertrochanteric fractures and identify independent risk factors.

**Methods:**

This retrospective study included patients with intertrochanteric fractures waiting for surgical interventions at our institution from June 2018 to December 2020. All patients received pharmacologic thromboprophylaxis after admission and ultrasound screening for DVT in both lower limbs before surgery. Demographic, clinical and laboratory data of these patients were collected to perform univariate analysis first. Subsequently, factors with a significant difference in univariate analysis were introduced into the multivariate logistic regression analysis to determine the independent risk factors for preoperative DVT.

**Results:**

A total of 266 patients were enrolled in this study. Seventy-one patients (26.7%) developed DVTs before surgery. The majority of DVTs were distal types (91.5%). There were 40 patients with DVTs only in the affected limb, 7 patients with DVTs only in the unaffected limb, and 24 patients with DVTs in both lower limbs. Advanced age, female, prolonged period from injury to admission, combined cranial trauma, shorter thrombin time, increased level of D-dimer and lower level of albumin proved to be the independent risk factors for preoperative DVT.

**Conclusions:**

We observed a high incidence of preoperative DVT in patients with intertrochanteric fractures. Identification of patients at high risk may improve the prevention and treatment of preoperative DVT in this population.

## Background

Hip fractures represent a major public health problem and are associated with increased disability, healthcare cost and mortality [1, 2]. Intertrochanteric fractures are one of the most common types of osteoporotic hip fractures. Patients with an advanced age account for the majority of this group, which also increases the risk of morbidity and mortality [3]. Patients with intertrochanteric fractures were commonly treated using surgical management by internal fixation. However, the incidence of perioperative complications remains high due to the characteristics of this population. Immobilization after intertrochanteric fracture results in venous stasis. Besides, patients with orthopedic trauma often have a hypercoagulable state and endothelial injury [4]. All three factors complete the Virchow Triad, which predispose these patients to deep vein thrombosis (DVT). DVT can lead to chronic venous insufficiency, post-thrombotic syndrome, and life-threatening pulmonary embolism, which are associated with increased hospitalization time, medical expense, patient unsatisfaction, morbidity and mortality [5]. Therefore, it is critical for clinicians to identify patients at high risk for DVT in this population. In this study, we aimed to determine the preoperative incidence of DVT in patients with intertrochanteric fractures by performing ultrasound screening and identify risk factors through multivariate regression analysis.

## Methods

This retrospective study was approved by the Institutional Review Board (IRB). We reviewed consecutive closed intertrochanteric fracture patients undergoing surgical interventions at our institution from June 2018 to December 2020. Patients with previous history of venous thromboembolism, pathological fractures, old fractures (more than three weeks before admission), long-term anticoagulant treatment, severe multiple injuries requiring several surgeries, contraindications for anticoagulants or coagulation disorders were excluded. All of the enrolled patients received pharmacologic thromboprophylaxis (rivaroxaban or low-molecular-weight heparin) after admission. Ultrasound screening for DVT in both lower limbs was performed one day before surgery. DVTs were classified into proximal DVTs and distal DVTs according to the location of thrombi. Proximal DVT is defined as the thrombus occurring proximally to the origin of the popliteal vein below the knee. Distal DVT refers to the thrombus located in the calf. Inferior vena cava (IVC) filter placement was only conducted in patients who were diagnosed with a proximal DVT. If patients had a distal DVT, planned surgeries were performed without additional preoperative interventions.

Medical records of these patients were reviewed to collect demographic and clinical information, including age, gender, body mass index (BMI), number of days between injury and surgery, number of days between injury and admission, number of days awaiting surgery, combined cranial trauma, blood transfusion before surgery, Caprini score, and preexisting illnesses (diabetes, hypertension, coronary heart disease and stroke). We also collected preoperative laboratory data of these patients, including hemoglobin (Hb) level, hematocrit (Hct), platelet count (PLT), albumin, estimated glomerular filtration rate (eGFR), C-reactive protein (CRP), plasma prothrombin time (PT), international normalized ratio (INR), activated partial thromboplastin time (APTT), thrombin time (TT), fibrinogen and D-dimer.

Statistical analysis was performed using STATA version 12.0 (Stata Corp. LP, College Station, TX, USA). In order to determine the multivariate regression model inputs, univariate analysis was conducted first. We compared the differences between DVT group and non-DVT group in demographic, clinical and laboratory characteristics. Qualitative variables were compared by a chi-square test, and quantitative variables were analyzed by a *t* test. Subsequently, factors with a significant difference (*P* value < 0.05) were introduced into the multivariate logistic regression analysis to determine the independent risk factors for preoperative DVT.

## Results

A total of 266 patients were enrolled in this study with a mean age of 74.5 ± 13.8 years (range 25–95 years). There were 107 males and 159 females. The mean number of days between injury and surgery was 6.8 ± 3.8 (range 1–25). The demographic, clinical and laboratory information of these patients is shown in Table 1.

**Table 1:** Patient’s demographic, clinical and laboratory information

Characteristics	Total (*n* = 266)
Gender	
Male (%)	107 (40.2)
Female (%)	159 (59.8)
Age, years (mean ± SD)	74.5 ± 13.8
BMI, kg/m^2^ (mean ± SD)	24.9 ± 4.1
Number of days between injury and surgery (mean ± SD)	6.8 ± 3.8
Combined cranial trauma (%)	15 (5.6)
Blood transfusion before surgery (%)	11 (4.1)
Caprini score (mean ± SD)	7.7 ± 1.1
Diabetes (%)	75 (28.2)
Hypertension (%)	132 (49.6)
Coronary heart disease (%)	41 (15.4)
Stroke (%)	28 (10.5)
PT, s (mean ± SD)	11.5 ± 0.8
INR (mean ± SD)	1.01 ± 0.07
APTT, s (mean ± SD)	27.4 ± 2.9
TT, s (mean ± SD)	16.9 ± 1.1
Fibrinogen, g/L (mean ± SD)	3.7 ± 1.1
D-dimer, mg/L (mean ± SD)	7.48 ± 5.99
eGFR, (mean ± SD)	100.9 ± 35.2
CRP, mg/L (mean ± SD)	43.9 ± 31.7
Albumin, g/L (mean ± SD)	36.9 ± 3.2
Hb, g/L (mean ± SD)	108.0 ± 19.7
Hct, % (mean ± SD)	32.0 ± 5.6
PLT, 10^9^/L (mean ± SD)	175.4 ± 67.9

SD, standard deviation; BMI, body mass index; PT, prothrombin time; INR, international normalized ratio; APTT, activated partial thromboplastin time; TT, thrombin time; eGFR, estimated glomerular filtration rate; CRP, C-reactive protein; Hb, hemoglobin; Hct, hematocrit; PLT, platelet count

Lower extremity DVTs were detected in 71 patients (26.7%). There were 40 patients with DVT only in the affected limb, 7 patients with DVT only in the unaffected limb, and 24 patients with DVTs in both lower limbs. Sixty-five patients with distal DVTs received planned surgery and prolonged postoperative anticoagulant therapy. Six patients were diagnosed with proximal DVTs and received IVC filter placement before surgery.

According to the univariate analysis, there were statistically significant differences between DVT group and non-DVT group in age (*P* < 0.001), gender (*P* = 0.001), number of days between injury and surgery (*P* = 0.002), number of days between injury and admission (*P* < 0.001), Caprini score (*P* < 0.001), combined cranial trauma (*P* = 0.016), thrombin time (*P* = 0.034), D-dimer (*P* = 0.012), albumin level (*P* < 0.001), Hb level (*P* = 0.005), and Hct (*P* = 0.013) (Table 2).

**Table 2:** Univariate analysis of the risk factors for preoperative DVT

Variables	Non-DVT (*n* = 195)	DVT (*n* = 71)	*P* value
Female gender (%)	105 (53.9)	54 (76.1)	0.001*
Age, years (mean ± SD)	72.7 ± 14.9	79.6 ± 8.4	< 0.001*
BMI, kg/m^2^ (mean ± SD)	24.9 ± 4.0	24.7 ± 4.5	0.679
Number of days between injury and surgery (mean ± SD)	6.4 ± 3.5	8.0 ± 4.5	0.002*
Number of days between injury and admission (mean ± SD)	2.7 ± 2.6	4.7 ± 4.0	< 0.001*
Number of days awaiting surgery (mean ± SD)	3.7 ± 2.4	3.3 ± 1.5	0.247
Combined cranial trauma (%)	7 (3.6)	8 (11.3)	0.016*
Blood transfusion before surgery (%)	6 (3.1)	5 (7.0)	0.151
Caprini score (mean ± SD)	7.5 ± 1.2	8.1 ± 0.9	< 0.001*
Diabetes (%)	56 (28.7)	19 (26.8)	0.754
Hypertension (%)	101 (51.8)	31 (43.7)	0.241
Coronary Heart Disease (%)	29 (14.9)	12 (16.9)	0.685
Stroke (%)	22 (11.3)	6 (8.5)	0.506
PT, s (mean ± SD)	11.5 ± 0.9	11.5 ± 0.7	0.905
INR (mean ± SD)	1.01 ± 0.08	1.01 ± 0.06	0.819
APTT, s (mean ± SD)	27.6 ± 3.0	27.1 ± 2.8	0.243
TT, s (mean ± SD)	17.0 ± 1.1	16.6 ± 1.0	0.034*
Fibrinogen, g/L (mean ± SD)	3.7 ± 1.0	3.9 ± 1.1	0.135
D-dimer, mg/L (mean ± SD)	6.93 ± 5.63	9.00 ± 6.69	0.012*
eGFR, (mean ± SD)	101.1 ± 34.9	100.5 ± 36.3	0.914
CRP, mg/L (mean ± SD)	43.9 ± 31.9	43.9 ± 31.1	0.997
Albumin, g/L (mean ± SD)	37.4 ± 3.3	35.5 ± 2.6	< 0.001*
Hb, g/L (mean ± SD)	110.1 ± 20.1	102.4 ± 17.4	0.005*
Hct, % (mean ± SD)	32.5 ± 5.7	30.6 ± 5.0	0.013*
PLT, 10^9^/L (mean ± SD)	171.1 ± 62.2	187.1 ± 81.0	0.091

DVT, deep vein thrombosis; SD, standard deviation; BMI, body mass index; PT, prothrombin time; INR, international normalized ratio; APTT, activated partial thromboplastin time; TT, thrombin time; eGFR, estimated glomerular filtration rate; CRP, C-reactive protein; Hb, hemoglobin; Hct, hematocrit; PLT, platelet count

*P < 0.05 was considered statistically significant

In order to determine the independent risk factors for preoperative DVT, factors with significant difference in univariate analysis were introduced into the multivariate logistic regression analysis. We found that advanced age (*P* = 0.003), female (*P* = 0.020), prolonged period from injury to admission (*P* = 0.028), combined cranial trauma (*P* = 0.043), shorter thrombin time (*P* = 0.029), increased level of D-dimer (*P* = 0.013) and lower level of albumin (*P* = 0.039) were the independent risk factors for preoperative DVT in patients with intertrochanteric fractures (Table 3).

**Table 3:** Multivariate logistic regression analysis to identify independent risk factors for preoperative DVT

Variables	OR	95% CI	*P* value
Female	2.40	1.15–4.99	0.020*
Age	1.07	1.02–1.11	0.003*
Number of days between injury and surgery	0.93	0.78–1.11	0.409
Number of days between injury and admission	1.26	1.03–1.55	0.028*
Combined cranial trauma	3.90	1.05–14.52	0.043*
Caprini score	1.01	0.68–1.48	0.978
TT	0.68	0.49–0.96	0.029*
D-dimer	1.07	1.01–1.13	0.013*
Albumin	0.88	0.78–0.99	0.039*
Hb	0.98	0.91–1.05	0.540
Hct	1.11	0.86–1.43	0.438

OR, odds ratio; CI, confidence interval; TT, thrombin time; Hb, hemoglobin; Hct, hematocrit

*P < 0.05 was considered statistically significant

## Discussion

In the present study, we identified a high incidence of preoperative DVT (26.7%) in patients with intertrochanteric fractures, even though prophylactic anticoagulation regimen was routinely applied before surgery. The majority of DVTs were distal types (91.5%), which only required prolonged postoperative anticoagulant therapy. Patients with proximal DVTs only accounted for a small portion of this population (8.5%). According to a previous study, the incidence of preoperative DVT in patients with intertrochanteric fracture was 37.61% and distal DVTs constituted 86.59% of all DVTs [6]. Zou et al. performed routine ultrasound Doppler scanning of bilateral lower extremities to detect admission DVT in the elderly with intertrochanteric fracture, and observed that 20.1% of these patients had DVT at the time of admission [7]. DVT is associated with chronic venous insufficiency, post-thrombotic syndrome, and life-threatening pulmonary embolism. Given the aggravation of DVT caused by surgery, preoperative evaluation of high-risk patients is strongly recommended. Besides, it is worth noting that 31 patients (43.7%) had DVT in the unaffected leg among the DVT group, which may be associated with the immobilization after fractures. Therefore, clinicians should pay more attention to this issue and encourage patients to start bedridden rehabilitation exercise immediately after injury.

This study identified prolonged period from injury to admission as an independent risk factor for preoperative DVT. Previous studies have also shown that delayed surgery is associated with a high risk of preoperative DVT [6, 8]. There are several common reasons leading to delayed surgery according to the previous study, including transfer from another hospital, late visit to the hospital, admission on weekend or holiday, drugs hold and patient’s medical condition requiring preoperative management [9]. Some organization factors causing delayed surgery may be avoidable, including admission on weekend or holiday, waiting for the results of preoperative tests, and unavailable operating room. It has been widely accepted that early surgery is beneficial to the patients with hip fractures, since surgery without delay proved to improve mobility, decrease early and late mortality, and reduce other medical complications [9–11]. However, this topic still remains controversial. Among the patients receiving early surgery, those with compromised medical conditions showed a high mortality rate [11]. Therefore, if patients have optimizable comorbid medical conditions contributing to mortality, surgery should be performed after stabilizing the patient’s condition to the greatest possible extent.

Most of the intertrochanteric fractures are caused by a fall, which is also the most common cause of traumatic brain injury in the elderly [12]. In this study, combined head trauma was found to be an independent risk factor for preoperative DVT in patients with intertrochanteric fractures. All of the patients with head trauma in this study only manifested mild symptoms, such as headache, a brief loss of consciousness and dizziness. None of them required surgery or medication for head trauma. Patients with a head trauma have proven to be at high risk of DVT [13, 14], which can be explained by several causative mechanisms. Head trauma leads to systemic inflammation, which promotes the release of von Willebrand factor (VWF) in endothelial cells [15]. VWF mediates platelet adhesion and subsequent thrombus formation [16]. Head trauma also causes the release of tissue factor from brain, which activates blood coagulation and secondary thrombosis [17].

In this study, a decreased level of albumin was found to be an independent risk factor for preoperative DVT. A low level of albumin is commonly regarded as an indicator of malnutrition. However, we did not find the relationship between low BMI and DVT in this study. An explanation for the association between albumin and DVT is that hypoalbuminemia may be a marker of inflammation, and inflammation can cause a hypercoagulable state in these patients [18]. Besides, hypoalbuminemia may be also caused by renal loss, which indicates the renal loss of anti-thrombotic proteins in the meantime [18]. Therefore, a low level of albumin can also serve as an indicator of hypercoagulable state [19].

In this study, the indications for IVC filter placement were based on the expert consensus of the Chinese Association of Orthopedics [20]. We only performed IVC filter placement in patients with proximal DVTs before surgery. If a patient was diagnosed with a distal DVT, the surgery was performed as planned without additional preoperative interventions. However, the indications for IVC filter placement in orthopedic patients with preoperative DVTs varied among medical centers. For patients with femur and hip fractures, Smith et al. conducted IVC filter placement in all patients diagnosed with preoperative DVTs and found that there were no related complications [8]. Otherwise, some studies employed indications similar to ours [21, 22]. However, there is no high-quality study comparing safety and efficacy among different indications. Therefore, further studies regarding this topic are warranted.

This study has several limitations. First, it should be noted that the present study has the limitations inherent to retrospective single-center study. Even though we attempted to include potential risk factors as many as possible in the analysis, there may be other confounders contributing to the preoperative DVT. Besides, the mean duration from injury to admission was relatively long in this study, which may increase the incidence of preoperative DVT.

## Conclusions

In this study, a high incidence of preoperative DVT (26.7%) was observed in patients with intertrochanteric fractures. Advanced age, female, prolonged period from injury to admission, combined cranial trauma, shorter thrombin time, increased level of D-dimer and lower level of albumin were found to be the independent risk factors for preoperative DVT. More attention about detecting and preventing preoperative DVT should be paid to patients with these risk factors.

## Data Availability

The datasets used and/or analyzed during the current study are available from the corresponding author on reasonable request.

## Abbreviations

DVT: Deep vein thrombosis
IRB: Institutional Review Board
IVC: Inferior vena cava
BMI: Body mass index
Hb: Hemoglobin
Hct: Hematocrit
PLT: Platelet count
eGFR: Estimated glomerular filtration rate
CRP: C-reactive protein
PT: Prothrombin time
INR: International normalized ratio
APTT: Activated partial thromboplastin time
TT: Thrombin time
VWF: Von Willebrand factor
